# Genetic Architecture of Hock Joint Bumps in Pigs: Insights from ROH and GWAS Analyses

**DOI:** 10.3390/ani15081178

**Published:** 2025-04-20

**Authors:** Lyubov Getmantseva, Maria Kolosova, Varvara Shevtsova, Anatoly Kolosov, Faridun Bakoev, Elena Romanets, Timofey Romanets, Siroj Bakoev

**Affiliations:** 1 Biotechnological Faculty, Don State Agrarian University, Persianovsky 346493, Russia; ilonaluba@mail.ru (L.G.); bakoevfaridun@yandex.ru (F.B.); lena9258@mail.ru (E.R.); timofey9258@mail.ru (T.R.); 2 All Russian Research Institute of Animal Breeding, Lesnye Polyany 141212, Russia; kolosov777@gmail.com (A.K.); siroj1@yandex.ru (S.B.); 3 Southern Scientific Centre of the Russian Academy of Sciences, Rostov-on-Don 344006, Russia; varvarashevtsova@gmail.com

**Keywords:** pigs, Large White breed, legs defects, ROH, GWAS, SNP

## Abstract

The genetic mechanisms underlying the formation of defects, such as bumps and growths on the hock joints in pigs, remain poorly understood. Therefore, the aim of this study was to investigate the relationship between runs of homozygosity (ROH) and the formation of hock joint bumps, with the goal of identifying associated SNPs and candidate genes involved in these defects. The study was conducted on a population of Large White breed pigs (n = 568) using runs of homozygosity (ROH) analysis and genome-wide association studies (GWAS). The results suggested that the predisposition to hock joint bumps in pigs may have arisen due to recent selective breeding pressure. Using GWAS, 27 SNPs were identified at the suggestive significance level, with one SNP (rs325478346) reaching genome-wide significance. These markers are localized in genes associated with various biological processes, including lipid metabolism (*VIPR2* and *CFTR*), inflammatory processes (*MTURN* and *ADCY2*), connective tissue structural integrity (*COL27A1*), muscle regeneration (*PAMR1*), and ion exchange and cellular homeostasis (*KCNIP4* and *NALCN*), as well as regulation of cell growth, extracellular matrix remodeling, and fibroblast differentiation (*CEP120* and *SCUBE3*). Further research utilizing omics technologies will provide deeper insights into the pathogenesis of hock joint bumps and contribute to the development of strategies for the prevention and potential treatment of this defect.

## Simple Summary

To investigate the genetic mechanisms underlying the formation of bumps on the hock joints in pigs, we employed runs of homozygosity (ROH) analysis and genome-wide association studies (GWAS). The aim of this study was to identify significant SNPs and candidate genes potentially contributing to the pathogenesis of bumps in the hock joint area. The results revealed that significant SNPs are localized in genes associated with lipid metabolism, inflammatory processes, connective tissue structural integrity, muscle regeneration, ion exchange, and cellular homeostasis. These findings contribute to a better understanding of the pathogenesis of bumps and provide a foundation for developing future pig breeding programs.

## 1. Introduction

Modern pig farming faces several pressing challenges, including meeting the growing demand for meat products, improving production efficiency, and enhancing the health and adaptive capabilities of animals under intensive farming conditions. The domestication of livestock for food, fat, leather, and fibers began approximately 12,000 years ago, marking the start of animal breeding [1]. Over centuries, pigs have been bred under diverse conditions worldwide. Advances in technology have led to the development of modern production systems with improved methods of housing, feeding, and breeding, significantly increasing animal productivity. However, intensive breeding programs focused on economically important traits can have negative consequences, such as reduced viability and the emergence of various defects in animals [2].

Limb defects represent a significant and widespread problem in modern pig farming, often leading to the culling of affected animals and posing a serious threat to the productivity and management of livestock industries [3]. Among these defects, the formation of bumps and growths in the hock joint area on the hind legs is particularly notable. These benign neoplasms, composed of connective tissue and free of pathogenic microflora, can occur in pigs of any age or weight [4]. Although they typically do not cause lameness, affected pigs often become unsuitable for sale, reducing the efficiency of breeding centers. Furthermore, these growths may be linked to underlying physiological processes and genetic predisposition, potentially impacting pig productivity and overall health [5].

Despite their economic and welfare implications, the genetic causes of hock joint bumps remain poorly understood. Previous studies have identified loci associated with the formation of these bumps in genes related to liver and kidney function, susceptibility to infections, and fatty acid composition [5,6]. Additionally, SNPs identified as potential fertility markers have been shown to influence the development of hock bumps [4]. However, a comprehensive understanding of the genetic architecture underlying this phenotype is still lacking.

Targeted breeding programs aimed at improving productivity often exacerbate health issues by directing metabolic pathways toward growth, reducing fatness, and increasing meat yield [7]. This highlights the need for a balanced approach that considers both productivity and animal health. Genome-wide association studies (GWAS) and runs of homozygosity (ROH) analysis are powerful tools for identifying genetic markers associated with complex traits, including disease resistance, reproduction, and meat quality [8,9,10,11,12,13]. These methods can also shed light on the genetic basis of hock joint bumps, providing insights into candidate genes and their potential roles in pathogenesis.

The goal of this study is to investigate the genetic factors underlying hock joint bumps in pigs. Using ROH and GWAS methods, we seek to identify homozygous genomic regions and associated SNPs linked to the formation of these bumps. By uncovering candidate genes and their potential roles, this research will contribute to a better understanding of the condition and inform future breeding programs to improve pig health and productivity.

## 2. Materials and Methods

### 2.1. Sampling

The studies were conducted on Large White breed pigs. The livestock population was established in 2005 using animals imported from an international breeding center, with subsequent development based on local genetic resources. The pig herd under study consisted of purebred animals from one of the leading breeding centers in the Russian Federation. A total of 568 animals (514 females and 54 males) were included in the study. All animals were maintained under the same environmental and management conditions. The breeding program is focused on improving key productive traits, including growth rate, feed conversion efficiency, and reproductive performance. Additionally, attention has been given to the meat quality and intramuscular fat content, which has influenced the population’s genetic structure. Genetic analysis has revealed signatures of artificial selection in genomic regions associated with these traits [14].

The “bumps/growths” phenotype is characterized by small formations in the hock joint area of the hind limbs in pigs. These formations are not accompanied by acute inflammation, redness, hyperthermia, or lameness. Histological examination revealed chronic tissue inflammation with areas of pathological changes [15]. The histological analysis reported the following findings: small hemorrhages; tissue edema; cellular infiltrates (including neutrophils, macrophages, lymphoid cells, plasmocytes, eosinophils, and histiocytes); proliferation of young connective tissue with fibroblasts and newly formed blood vessels; areas of mature connective tissue with an increased number of collagen fibers; and regions of coarse fibrous tissue with dense collagen fibers. The phenotype was visually assessed in pigs aged 45–60 days and categorized as follows: “1”—absence of bumps (331 females and 38 males) and “2”—presence of bumps (183 females and 16 males).

Tissue samples for DNA isolation and genotyping were collected by veterinary specialists at the farm following standard operating procedures.

### 2.2. Genotyping and Quality Control

The samples were genotyped using the GeneSeek^®^ GGP SNP80x1_XT array (Illumina Inc., San Diego, CA, USA). Quality control (QC) was performed using PLINK v1.9 [16] to remove individuals with more than 10% missing genotypes (--mind 0.1), SNPs with more than 10% missing values (--geno 0.1; 1458 variants removed), and SNPs with a minor allele frequency (MAF) of less than 0.05 (--maf; 11,042 variants removed). After QC, 58,550 SNPs remained for further analysis. Population structure was determined using the principal component analysis (PCA) method in PLINK v1.9 [16] (see Supplementary Materials Figure S1). To identify runs of homozygosity (ROH), the following filters were applied: --geno 0.1, --mind 0.1, and --maf 0.05. For GWAS analysis and PCA, linkage disequilibrium (LD) pruning was performed using the following parameters: a window size of 50 kb, a step size of 5, and an r^2^ threshold of 0.5 (--indep-pairwise 50 5 0.5).

### 2.3. Homozygosity Analysis (ROH)

Homozygosity analysis was performed using the RZooRoH package in R version 0.3.2.1 [10]. The Bayesian approach of full probabilistic modeling was used to identify ROH segments (HBD—homozygosity by descent, which refers to inherited regions of autozygosity where identical haplotypes are inherited from a common ancestor) [17,18]. The segments were divided into classes with R = 2, 4, 8, 16, 32, 64, 128, 256, and 512. Each class indicates the time when the regions of autozygosity were inherited from ancestors (R × 0.5). For example, Class 2 HBDs are inherited from parents (2 × 0.5), Class 4 from grandparents (4 × 0.5), and so on. The individual degree of inbreeding was defined as the total HBD (the sum of all HBD classes).

The criteria for determining the top HBD regions were (1) the frequency of HBD in the group being at least 80% and (2) the presence of at least 4 SNPs. To analyze the association of autozygosity with the formation of hock joint bumps, pigs were divided into two groups: LW1 (without bumps) and LW2 (with bumps). The proportion of the genome covered by HBD in each class was calculated separately for each group. The statistical significance of the differences between the groups was assessed using a *t*-test. Additionally, a logistic mixed model was used to analyze the relationship between the level of homozygosity in each class and the presence of bumps. The model included the following components: binary phenotype: healthy (1) or sick (2) and basic responsibility *l_i_*, which follows a normal distribution. If *l_i_* exceeds the threshold t, then *y_i_* = 2 (sick); otherwise, *y_i_* = 1 (healthy). Fixed effects were season–herd–year and sex.

### 2.4. Heritability

Heritability of the “bumps” trait was estimated using the Restricted Maximum Likelihood (REML) method implemented in the Genome-wide Complex Trait Analysis (GCTA) software 1.94.1 [19,20]. This approach is widely used to estimate genetic variance and heritability for complex traits. Genetic variance (V(G)) and total phenotypic variance (Vp) were calculated, and heritability was determined as the ratio of genetic variance to total phenotypic variance (h^2^ = V(G)/Vp). The REML method accounts for the genetic structure of the population by incorporating the genomic relationship matrix (GRM), which minimizes the influence of familial relationships on heritability estimates.

### 2.5. GWAS

For the GWAS (Genome-Wide Association Study), the Mixed Linear Model Association (MLMA) was used in the GCTA program 1.94.1 [19,20]. To avoid false-positive associations due to population structure, a genetic relationship matrix (GRM) was incorporated into the model. The GRM accounts for the sample structure across the entire genome. The MLMA model also included the GRM and sex (as a fixed effect) to evaluate the variance components, SNP heritability, and association statistics. To control for false positives during the GWAS, a false discovery rate (FDR) correction was applied, with threshold values set at 0.01 for genome-wide significance and 0.05 for suggestive significance. The threshold *p*-values were determined using the formula:

*P* = *FDR* × *N/M*,

where *FDR* (false discovery rate) = 0.01 (genome-wide significance level) or 0.05 (suggestive significance level); *N* = number of variants with *P <* FDR; *M* = total number of variants tested. The GWAS results were visualized using Manhattan plots, which display *−*log10*(P)* for each SNP across the genome.

### 2.6. Functional Analysis

The functional roles of the identified genes associated with significant SNPs were investigated using data from PMC (PubMed Central) and other literature reviews. Genes with functional significance for the trait under study were selected as candidate genes.

## 3. Results and Discussion

### 3.1. Autozygosity Analysis

**Figure 1:**
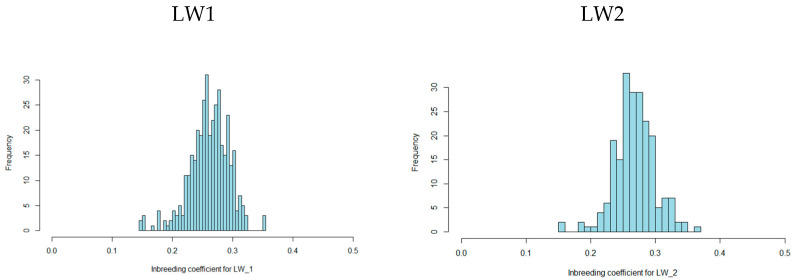
Distribution of the inbreeding coefficient in LW1 and LW2.

According to the genome scanning results, the total proportion of autozygosity in the LW1 and LW2 groups was 0.26 and 0.27, respectively. The minimum values were the same in both groups, approximately 0.15, while the maximum values were higher in the LW2 group (0.37) compared to the LW1 group (0.35) (Figure 1).

**Figure 2:**
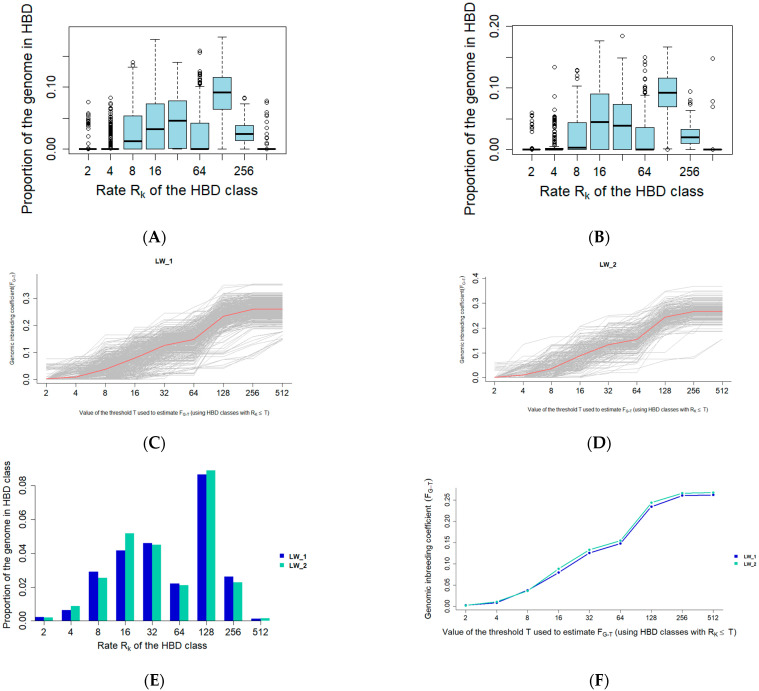
Autozygosity distribution by class in the LW1 and LW2 groups: (**A**) the autozygosity distribution by class in LW1; (**B**) the autozygosity distribution by class in LW2; (**C**) autozygosity distribution by class in LW1 at the individual level; (**D**) autozygosity distribution by class in LW2 at the individual level; (**E**) autozygosity distribution by class in different classes in LW1 and LW2; (**F**) the cumulative curve of the autozygosity regions in LW1 and LW2.

The distribution of HBD by class corresponds to the general trend observed in modern pigs. HBD_128 represents the largest proportion of autozygosity, while HBD_4 and HBD_512 represent the smallest proportions. The HBD_2 segments are practically absent in both the LW1 and LW2 groups (see Supplementary Materials Table S1). Figure 2 illustrates the distribution of autozygosity across the corresponding classes in the LW1 and LW2 pig genomes.

**Figure 3:**
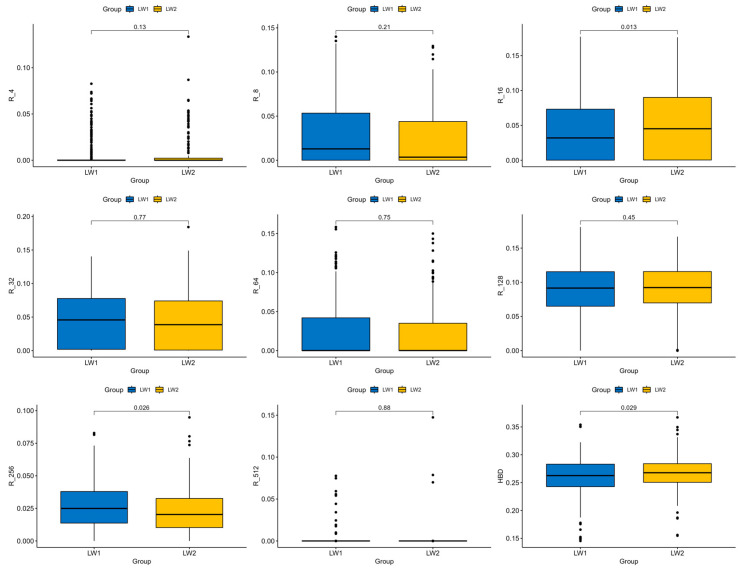
Differences in autozygosity in groups LW1 and LW2.

In general, the level of autozygosity differs slightly between the two groups (Figure 3). The analysis revealed that the total autozygosity level in LW2 pigs is 0.006 higher (*p* = 0.029) compared to LW1 pigs. Additionally, the autozygosity level in LW2 pigs is significantly higher in class R_16 (*p* = 0.013) and significantly lower in class R_256 (*p* = 0.026).

Estimates of the effects of the homozygosity levels on the bump phenotype using a logistic mixed model showed similar results. The effect of R_16 is statistically significant and positive (*p* = 0.046), while the effect of R_256 is statistically insignificant and negative (*p* = 0.074). Interestingly, an increase in R_16 autozygosity was associated with a positive effect on bump formation in pigs, whereas regions of R_256 autozygosity showed a potential tendency toward resistance to the bump phenotype. This suggests that recent breeding pressure may influence the predisposition to bumps in pigs.

**Figure 4:**
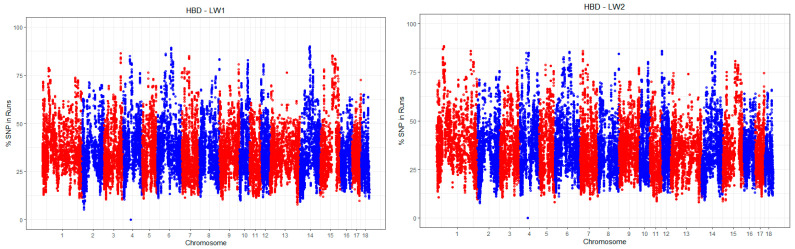
The frequencies of SNPs localized in the homozygous regions in pigs LW1 and LW2.

It is likely that the influence of autozygosity on productivity traits and resistance to various diseases is determined not only by the proportion of autozygosity but also by the characteristics of the segments and their genomic locations. Figure 4 illustrates the frequencies of SNPs localized within regions of homozygosity.

The most common HBDs identified in the two groups are presented in Table S2 (see Supplementary Materials). The analysis revealed seven HBDs on chromosomes SSC1, 4, 7, and 14, which are characteristic only of LW2 pigs with the “bumps” phenotype. These regions contain genes associated with the regulation of cellular signals (*PKIA*, *NCOA4*, *MARCHF8*, and *PKA*); synthesis and transport of neurotransmitters (*CHAT*, *SLC44A1*, and *SLC18A3*); lipid metabolism; magnesium homeostasis and energy balance (*ABCA1*, *TRPM6*, *OGDHL*, and *PARG*); stress response; regulation of protein degradation; DNA repair (*OGDHL*, *PARG*, and *NIPSNAP3A*); and immune responses and inflammation (*RORB*, *MARCHF8*, and *PKIA*).

### 3.2. Heritability of the Trait Evaluation

The REML (Restricted Maximum Likelihood) analysis was performed to assess the heritability of the trait. The results showed that the genetic variance (V(G)) was 0.08 (SE = 0.02) and the total phenotypic variance (Vp) was 0.22 (SE = 0.01). The heritability of the “bumps“ trait, calculated as h^2^ = V(G)/Vp), was estimated at 0.35 (SE = 0.08) (Table 1). This indicates that 35.04% of the variation in the trait is influenced by genetic factors. These findings suggest potential opportunities for improving the trait through genomic selection.

### 3.3. GWAS Results

**Figure 5:**
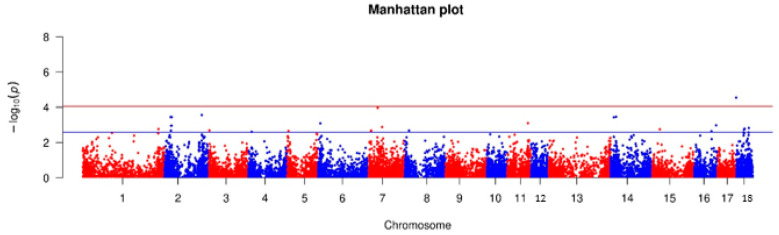
Manhattan plot for bumps in the hock joints on the hind limbs of pigs.

The Genome-Wide Association Analysis (GWAS) identified 27 SNPs that reached the suggestive significance level. Among these, one SNP (rs325478346) achieved genome-wide significance (Figure 5). These SNPs encompassed various types of genetic variants, including intergenic variants (n = 14), intron variants (n = 11), a 3′ UTR variant, and a regulatory region variant.

The GWAS results serve as a valuable tool for comparing phenotypically associated SNPs with genes or identifying variants in genes encoding proteins which functions may be directly or indirectly involved in the pathogenesis of the bumps phenotype (Table 2).

The most significant association was found for SNP rs325478346, located on chromosome 18 in the VIPR2 gene. This gene encodes vasoactive intestinal peptide receptor 2, also known as VPAC2R, a membrane protein that regulates insulin secretion, muscle mass, lipid metabolism, and circadian rhythms. *VIPR2* plays a crucial role in maintaining muscle mass, particularly fast-twitch fibers, and its deficiency is associated with stunted growth and altered IGF-I levels [21]. In adipocytes, this receptor regulates lipid metabolism, and its knockout leads to a reduction in fat mass and a compensatory increase in muscle mass [22]. Additionally, the *VIPR2* is involved in thermoregulation and the control of circadian rhythms, influencing energy homeostasis and physiological processes [23]. Dysfunction of the *VIPR2* can result in growth retardation, changes in IGF-I levels, and disorders of lipid metabolism. These processes may contribute to the development of bumps, as one of the potential causes of their formation is impaired lipid metabolism and chronic inflammation.

The study also highlights the *CFTR* gene, which encodes a protein that functions as a chloride ion channel across cell membranes. This gene plays a key role in maintaining the water–ion balance in body tissues. Dysfunction of the *CFTR* is associated with pathologies such as cystic fibrosis, characterized by chronic respiratory tract infections, digestive disorders, and abnormalities in bone and lipid metabolism [24,25,26]. In pigs, dysfunction of the *CFTR* may lead to an imbalance in fatty acids, which can manifest at birth and contribute to the development of inflammatory processes and other pathologies.

Histological analysis of bumps in the hock joint region of swine revealed signs of chronic low-grade tissue inflammation without evidence of an acute phase, angiogenesis, cellular infiltration, and the formation of connective tissue structures at varying stages of maturity [15]. The identified SNPs are located in genes that regulate inflammatory processes, cell differentiation, angiogenesis, and fibrosis.

The rs337584442 SNP is located in the *MTURN* gene, which encodes a protein that suppresses the NF-κB signaling pathway, playing a key role in regulating inflammation [27]. The rs339814189 SNP is located in the *ADCY2* gene, which encodes an enzyme involved in the synthesis of cyclic adenosine monophosphate (cAMP) from ATP. Dysregulation of this gene can lead to an imbalance between cell proliferation and differentiation, affecting inflammation and angiogenesis [28]. Additionally, cAMP imbalance can alter the immune response, exacerbating inflammation and contributing to the pathological process. The rs80793012 SNP is located in the *NALCN* gene, which encodes a sodium channel that regulates membrane potential and cell excitability, including in neurons and smooth vascular muscle cells. Dysfunction of the *NALCN* can disrupt ionic homeostasis, affecting cell proliferation, inflammation, and angiogenesis. The *NALCN* is also associated with several diseases, such as bipolar disorder, schizophrenia, Alzheimer’s disease, and heart disease [29]. In the context of bumps in pigs, the *NALCN* may play a role in regulating vascular tone and microcirculation, contributing to chronic inflammation and altered tissue vascularization. Disruptions in ion transport can also activate fibroblasts and remodel the extracellular matrix, leading to fibrosis and the thickening of connective tissue.

The rs81403653 SNP is located in the *KCNIP4* gene, which encodes a protein that interacts with voltage-gated potassium channels. This gene is involved in regulating neurotransmitter release, smooth muscle contraction, heart rate, and insulin secretion. In humans, SNPs in this gene are associated with neurological disorders [30], cancer [31], and chronic kidney disease [32]. In animals, *KCNIP4* has been identified as an important quantitative trait locus (QTL) region associated with growth traits. Dysfunction of the *KCNIP4* can lead to disruptions in the ion balance, destabilization of the cellular membrane potential, and excessive proliferation of connective tissue. Significant associations have been found in chicken populations [33,34], beef cattle [35], meat rabbits [36], and sheep [37]. The association of *KCNIP4* with QTLs regulating growth in animals, as well as with various diseases in humans, makes it a potentially significant candidate in the pathogenesis of bumps in pigs.

The rs81351938 SNP is located in the *COL27A1* gene, which encodes a crucial component of the extracellular matrix. This protein provides strength and elasticity to tissues, particularly cartilage and skin, and is involved in the formation and maintenance of the matrix structure. *COL27A1* plays a key role in endochondral ossification, regulating bone growth, and influences cell adhesion, migration, and differentiation, which are essential for normal tissue function [38]. Dysfunction of this gene can lead to inflammation, fibrosis, and the formation of bumps, especially in areas subjected to high mechanical stress, such as the hock joints in pigs.

The rs81327279 SNP is located in the *PAMR1* gene, which encodes a protein involved in muscle tissue regeneration and the regulation of inflammatory processes. In both humans and animals, *PAMR1* plays an important role in repairing damaged muscles and may be involved in intercellular signaling. Dysregulation of this gene is associated with progressive muscular dystrophy and certain types of cancer. *PAMR1* functions as a tumor suppressor gene, and its overexpression has been shown to suppress cell growth [39]. In the study by Qi et al. [40], it was demonstrated that the expansion of capillaries and the disorganization of collagen fibers are associated with the height of the crest in roosters. The authors suggested that reduced expression of the *PAMR1* gene may inhibit the development of the crest [40]. In the context of bump formations in pigs, PAMR1 may contribute to impaired extracellular matrix remodeling, chronic inflammation, and insufficient tissue regeneration, leading to excessive fibrosis.

The rs81296219 SNP is located in the *CEP120* gene, which encodes a protein involved in the formation of centrioles and primary cilia. These structures play a key role in intercellular signaling, cell polarity, and morphogenesis. Dysregulation of *CEP120* is associated with abnormalities in the development of bone and cartilage tissues. In the pathogenesis of pineal formations in pigs, *CEP120* may influence the activity of fibroblasts and osteoblasts, promoting the proliferation of connective tissue and altering bone formation processes [41]. Defects in centrosome organization can lead to an imbalance in proliferation and inflammatory responses, exacerbating the pathological process and disrupting tissue regeneration. This can result in the formation of fibrous bone structures in the affected areas.

The rs80937427 SNP is located in the *SCUBE3* gene, which encodes a secreted protein involved in regulating cell proliferation, angiogenesis, inflammation, and tissue remodeling through the activation of the TGF-β/BMP signaling pathways. *SCUBE3* acts as a co-receptor for BMP2/BMP4, enhancing their signaling pathways and promoting osteogenesis and chondrogenesis [42]. Loss of the *SCUBE3* function can lead to hereditary developmental disorders of the skeleton and connective tissue due to defects in BMP signaling. This gene is also involved in regulating inflammatory processes and may influence tumor development by modulating cell proliferation and intercellular interactions. Disruption of its activity can impair wound healing and tissue repair, making it a critical factor in homeostasis and morphogenesis. Liu et al. [43] suggested a potential association of *SCUBE3* with skeletal development, tissue repair, and growth potential in pigs. In the context of bumps in pigs, *SCUBE3* may play a key role by influencing inflammation, angiogenesis, and connective tissue proliferation. Its involvement in extracellular matrix remodeling and fibroblast differentiation may contribute to excessive fibrosis and chronic inflammation. Active vascular proliferation in the affected areas may be linked to *SCUBE3’s* role in angiogenesis, which sustains long-term inflammation.

The development of hock bumps in pigs is influenced by both environmental conditions and individual physiological characteristics. While the exact mechanisms remain incompletely understood, genetic predisposition appears to play a significant role. This predisposition affects lipid and fatty acid metabolism, the regulation of inflammatory processes, extracellular matrix remodeling, and tissue regeneration.

Disruptions in fatty acid metabolism, liver dysfunction, infections, or injuries can initiate inflammatory processes. Mechanical stress on the hocks, particularly under conditions of intensive fattening or restricted mobility, increases the vulnerability of these tissues to structural changes. Chronic inflammation promotes the growth of atypical tissues and their abnormal proliferation. Fibrosis further exacerbates mechanical stress, activating immune cells (e.g., macrophages and lymphocytes), which, in turn, sustains chronic inflammation, creating a self-perpetuating cycle. Genetic factors likely modulate key signaling pathways involved in lipid metabolism, inflammatory responses, connective tissue integrity, muscle regeneration, ion exchange, and cellular homeostasis. Collectively, these processes contribute to the formation of hock bumps in pigs.

Further research utilizing a wide range of omics technologies will enable a deeper exploration of the pathogenesis of hock bumps, elucidate the regulatory mechanisms underlying inflammatory and reparative processes, and facilitate the development of effective prevention and treatment strategies. The study could also benefit from incorporating advanced microfluidic-based sorting methods, which have proven invaluable in genetic research for precise phenotypic classification and the isolation of specific cell populations [44]. By integrating these cutting-edge approaches, researchers can achieve a more comprehensive understanding of the molecular and cellular dynamics driving this condition, ultimately paving the way for targeted interventions.

## 4. Conclusions

The formation of bumps in pigs is influenced by a combination of genetic, physiological, and environmental factors. Selective breeding pressures have likely contributed to metabolic changes, including altered fatty acid metabolism and an increased susceptibility to chronic inflammation. Regions of autozygosity inherited approximately eight years ago (R_16) are associated with bump formation, whereas older regions (R_256, over 120 years) may indicate resistance. A Genome-Wide Association Study (GWAS) identified 10 SNPs in genes linked to lipid metabolism (*VIPR2* and *CFTR*), inflammation (*MTURN* and *ADCY2*), connective tissue integrity (*COL27A1*), muscle regeneration (*PAMR1*), ion transport (*KCNIP4* and *NALCN*), and extracellular matrix regulation (*CEP120* and *SCUBE3*). These findings provide valuable genetic markers for breeding programs aimed at reducing bump formation in pigs.

## Figures and Tables

**Table 1:** Variance components and heritability estimates for hock joint bumps in pigs.

Source	Variance	SE
V(G)	0.08	0.02
V(e)	0.15	0.02
Vp	0.22	0.01
V(G)/Vp	0.35	0.08

**Table 2:** SNPs localized in genes.

Chr	SNP	Variant	Gene
1	rs81351938	intron variant	*COL27A1*
2	rs81296219	3 prime UTR variant	*CEP120*
2	rs81327279	intron variant	*PAMR1*
7	rs80937427	intron variant	*SCUBE3*
8	rs81403653	intron variant	*KCNIP4*
11	rs80793012	intron variant	*NALCN*
16	rs339814189	intron variant	*ADCY2*
18	rs81297167	intron variant	*CFTR*
18	rs337584442	intron variant	*MTURN*
18	rs325478346	intron variant	*VIPR2*

## Data Availability

Data are available upon reasonable request.

## Supplementary Materials

The following supporting information can be downloaded at https://www.mdpi.com/article/10.3390/ani15081178/s1: Table S1: The proportion of the genome in various HBD classes; Table S2: Best HBDs identified in two groups of pigs; Figure S1. Graphical representation of the principal component analysis (PCA).
