# Supplementary material for: Genetic Architecture of Hock Joint Bumps in Pigs: Insights from ROH and GWAS Analyses
Source: Animals (Basel). 2025 Apr 20;15(8):1178. doi: 10.3390/ani15081178 (PMC12023961; doi:10.3390/ani15081178)
Supplement: Supplementary file 1 [file animals-15-01178-s001.zip › animals-3517952-supplementary.pdf]

Table S1. The proportion of the genome in various HBD classes.

Class	LW1					LW2				
	mean	Se	Sd	min	max	mean	se	sd	Min	max
R_4	0,0064	0,0008	0,0150	0,0000	0,0827	0,0088	0,0013	0,0192	0,0000	0,1335
R_8	0,0291	0,0018	0,0345	0,0000	0,1400	0,0255	0,0023	0,0331	0,0000	0,1294
R_16	0,0417	0,0022	0,0428	0,0000	0,1771	0,0518	0,0034	0,0491	0,0000	0,1762
R_32	0,0460	0,0020	0,0382	0,0000	0,1405	0,0450	0,0029	0,0424	0,0000	0,1843
R_64	0,0221	0,0018	0,0355	0,0000	0,1583	0,0211	0,0024	0,0351	0,0000	0,1500
R_128	0,0866	0,0021	0,0409	0,0000	0,1808	0,0892	0,0026	0,0374	0,0000	0,1668
R_256	0,0261	0,0009	0,0177	0,0000	0,0828	0,0227	0,0012	0,0172	0,0000	0,0949
R_512	0,0013	0,0004	0,0082	0,0000	0,0776	0,0014	0,0009	0,0125	0,0000	0,1474
HBD	0,2615	0,0017	0,0327	0,1453	0,3539	0,2675	0,0021	0,0310	0,1548	0,3671

Table S2. Best HBDs identified in two groups of pigs.

Group	Chromosome	First SNP	Last SNP
LW1	3	115716410	116455937
LW1	4	44834041	48454448
LW1	4	58820931	59697295
LW1	6	57859279	58432994
LW1	6	97620286	98870990
LW1	6	108956439	109639091
LW1	7	49887262	50205289
LW1	8	137884266	133644258
LW1	10	57123236	57213712
LW1	10	57325327	57593745
LW1	12	16431592	16734134
LW1	14	66346934	68041458
LW1	14	69492065	70250940
LW1	14	71116678	72180687
LW1	15	82602581	84114197
LW1	15	100767413	103751874
LW2	1	40917288	42669178
LW2	1	47795449	48272677
LW2	1	227132260	227957091
LW2	1	246116668	246411573
LW2	4	57631177	57904163
LW2	4	58820931	59697295
LW2	6	57859279	58817365
LW2	6	97787540	98778423
LW2	7	17835129	18582471
LW2	8	137884266	138930735
LW2	12	1610942	2113332
LW2	14	66411511	67269867
LW2	14	71116678	71463045
LW2	14	90110669	90856487
LW2	15	83231112	83971851

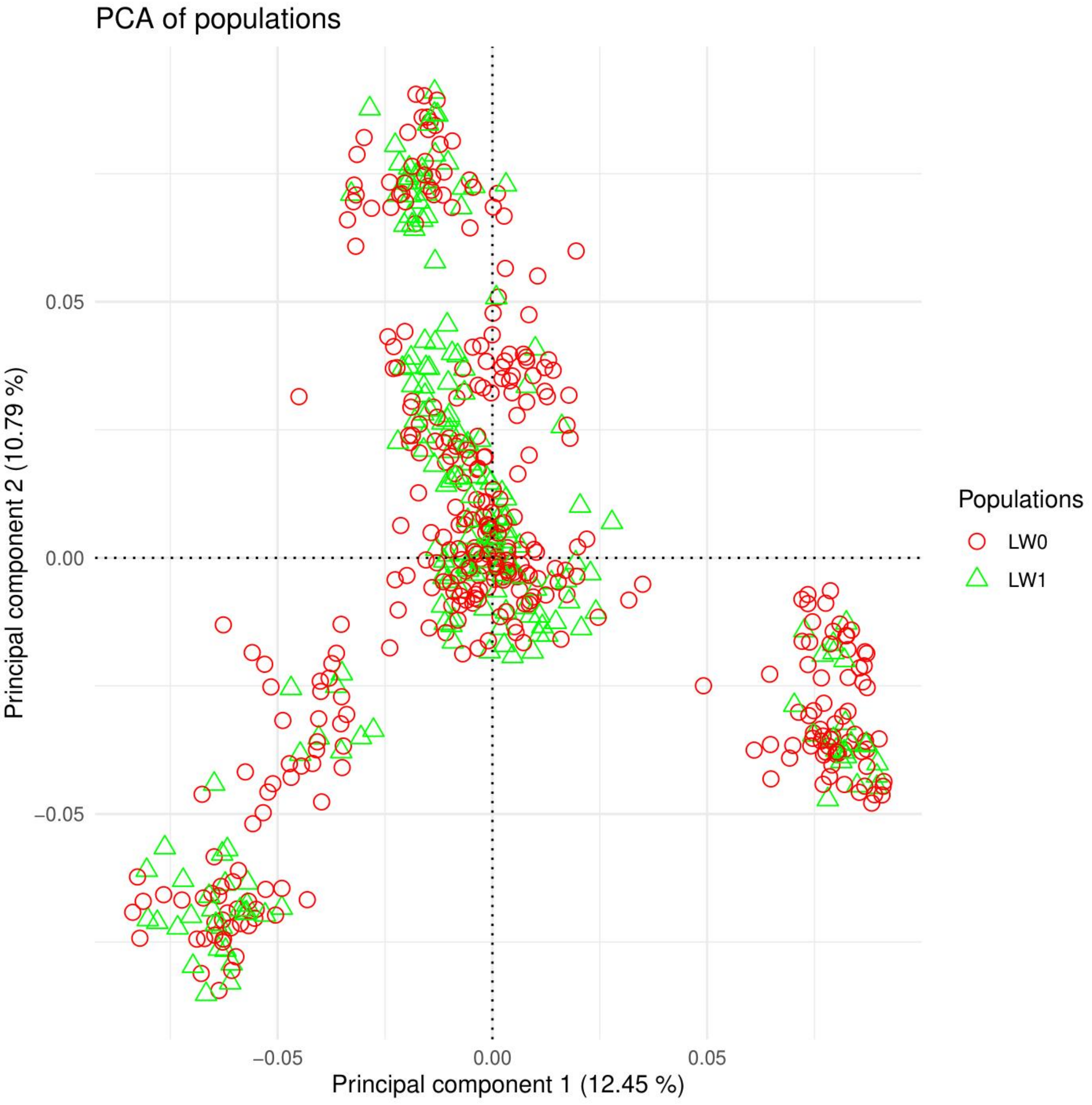

Figure S1. Graphical representation of the principal component analysis (PCA)
